# A Voice for Research, a Voice for Patients

**DOI:** 10.1371/journal.pbio.0020182

**Published:** 2004-06-15

**Authors:** Daniel Perry

## Abstract

In response to the Blackburn and Rowley essay on the President's Council on Bioethics, several thought-provoking opinions on ethical challenges in biomedical research are expressed by prominent stakeholders

In the very thoughtful essay “Reason as Our Guide” by Drs. Elizabeth Blackburn and Janet Rowley (2004), the authors highlight a key concern with the reports published by the President's Council on Bioethics—the lack of credible scientific information being passed on to policy makers.

Blackburn and Rowley point out many areas of the report “Monitoring Stem Cell Research” that needed correction from a scientific standpoint. While it is impossible to include every suggestion in a report that seeks to draw consensus from a large panel of members, in a heated, political debate like that surrounding embryonic stem cell research and therapeutic cloning, providing the most accurate and complete scientific information to policy makers is crucial. Unfortunately, with the recent dismissal of Dr. Blackburn from the Council, there will now be one less voice for scientific research and for the potential the research holds for curing disease and alleviating the suffering of millions.

Speaking for the Coalition for the Advancement of Medical Research, our concern is not only the small number of researchers on the Council and lack of complete scientific data being shared with policy makers, but the absence of patient representation on the Council itself. With the exception of public comment periods, patient organizations have no voice in the work of the Council as it discusses issues that profoundly impact them. Now, with one less member standing up for research and thus patients, our concern grows even stronger.

The Blackburn and Rowley essay also correctly points out that there is more published work on adult stem cell research because of a “paucity of funding for research using embryonic stem cells.” Despite this lack of federal and private funding, advances continue to be made—but just think of the advances we could have had if only there were a supportive federal policy that encouraged embryonic stem cell research instead of stifling it. We hope—in light of scientific advances made over the past several years and the strong support of the scientific community, including the National Institutes of Health, the Health and Human Services Department, and the National Academy of Sciences—that the President will reevaluate the current federal policy for stem cell research and consider easing the restrictions.

We commend Drs. Blackburn and Rowley for trying to set the record straight in their essay, and applaud their efforts to stand up for medical research, which has the potential to benefit us all.
